# Combined neat model for the prognosis of postoperative stage III‐N2 non‐small cell lung cancer

**DOI:** 10.1111/1759-7714.13585

**Published:** 2020-07-29

**Authors:** Yirui Zhai, Zhouguang Hui, Yu Men, Yang Luo, Yushun Gao, Jingjing Kang, Xin Sun, Jianyang Wang

**Affiliations:** ^1^ Department of Radiation Oncology National Cancer Center/National Clinical Research Center for Cancer/Cancer Hospital, Chinese Academy of Medical Sciences & Peking Union Medical College Beijing China; ^2^ Department of VIP Medical Services National Cancer Center/National Clinical Research Center for Cancer/Cancer Hospital, Chinese Academy of Medical Sciences and Peking Union Medical College Beijing China; ^3^ Department of Medical Oncology National Cancer Center/National Clinical Research Center for Cancer/Cancer Hospital, Chinese Academy of Medical Sciences & Peking Union Medical College Beijing China; ^4^ Department of Thoracic Surgery National Cancer Center/National Clinical Research Center for Cancer/Cancer Hospital, Chinese Academy of Medical Sciences & Peking Union Medical College Beijing China

**Keywords:** CA 125, CEA, CYFRA 21‐1, non‐small cell lung cancer, serum tumor marker

## Abstract

**Background:**

Lung cancer serum tumor markers including carcinoembryonic antigen (CEA), cytokeratin 19 fragment (CYFRA21‐1), and carbohydrate antigen 125(CA125) as prognostic predictors is controversial. Therefore, this study aimed to evaluate the association between these markers and the survival of patients with postoperative stage III‐N2 non‐small cell lung cancer (NSCLC).

**Methods:**

We enrolled 1011 patients with pathologically confirmed stage III‐N2 NSCLC who underwent resection and whose pretreatment serum tumor marker levels were available. Patients were categorized according to their serum levels into low‐, medium‐, and high‐risk groups. Overall survival (OS), progression‐free survival (PFS), local regional relapse‐free survival (LRFS), and distant metastasis‐free survival (DMFS) were calculated from the date of resection. Their association with each serum tumor marker was assessed using the log‐rank test.

**Results:**

Abnormal CEA levels were associated with worse five‐year OS, PFS and DMFS; abnormal CYFRA21‐1 levels were associated with worse five‐year OS and LRFS; and abnormal CA125 levels were associated with worse five‐year OS, PFS, LRFS and DMFS. Among the risk groups, there were significant differences in five‐year OS, PFS, LRFS and DMFS (*P* = 0.000). In propensity score matching analysis, the model also achieved prognostic significance for all four survival classifications (*P* = 0.001–0.004) among the three risk groups.

**Conclusions:**

The combined model achieved prognostic significance for all survival outcome types. The serum tumor markers tested are useful prognostic predictors for postoperative NSCLC patients but not for all survival outcomes. The combination of the three indices is more reliable in predicting all four of the survival outcomes.

**Key points:**

**Significant findings of the study:**

Serum CEA, CYFRA21‐1, and CA125 levels can be used as prognostic factors of postoperative N2 non‐small cell lung cancer patients but not for all survival outcomes, suggesting that combinative detection of all three indices would be more reliable.

**What this study adds:**

Our model utilizes available technology, with conventional cutoff values, inexpensive costs, and simple mathematics methods and, thus, can be feasibly employed by clinicians or oncologists.

## Graphical abstract

From a large number of patients' analysis, we achieved a neat model to be able to predict the OS, PFS, LRFS and DMFS of postoperative III‐N2 NSCLC.

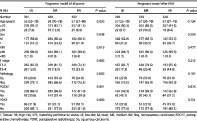

## Introduction

Non‐small cell lung cancer (NSCLC) accounts for 85% of lung cancers.^1^ Approximately 30% of patients with NSCLC are diagnosed first at stage III of the disease.^2^ In such cases, surgery is the first treatment of choice, although postoperative therapeutic regimens are controversial. Moreover, response to treatment is heterogeneous. Several studies have attempted to determine pretreatment prognostic factors to help plan treatments.

Studies have used circulating tumor cells and cell‐free tumor DNA and RNA as a basis for predicting patient survival and recurrence, but their utilization is limited owing to the testing complexity and high costs.^3^ To solve these problems, constructing a convenient and effective model for prognosis prediction is of great significance.

Serum tumor markers for lung cancer including carcinoembryonic antigen (CEA), cytokeratin 19 fragment (CYFRA 21‐1), carbohydrate antigen 125 (CA 125), squamous cell carcinoma antigen (SCC), neuron specific enolase (NSE), and progastrin releasing peptide (ProGRP) are widely used for diagnosis, but their role as prognostic predictors is still debated. Among them, SCC, NSE, and ProGRP are strongly associated with certain histological types while the other three are not.^4^ This study was designed to gain a better insight into the relationship between the serum tumor markers CEA, CYFRA 21‐1, and CA 125 and the survival outcomes of patients with stage III‐N2 NSCLC after R0 resection.

## Methods

### Ethics

This study conformed to the provisions of the Declaration of Helsinki, and it was approved by the ethics committee of the National Cancer Center, China, in 2016 (approval number NCC2016 YL‐04). Informed patient consent was obtained before treatments.

### Patient selection

Patient eligibility criteria were as follows: patients ≥ 18 years with pathologically confirmed operable stage III‐N2 NSCLC (according to the American Joint Committee of Cancer seventh staging system), underwent R0 resection in our institution, available serum CEA, CYFRA 21‐1, and CA 125 pretreatment data, full medical documents, and follow‐up records. The exclusion criteria were as follows: pregnancy, breastfeeding, other malignancies except nonmelanoma skin cancer or cervical carcinoma in situ, clinical evidence of infection, and autoimmune disease. Data on demographics, pathological subtypes, postoperative treatments, and survival were collected.

### Specimens and laboratory tests

Serum CEA, CYFRA 21‐1, and CA 125 were tested using immunoelectrochemistry. The records were taken from the database. The normal ranges of CEA, CYFRA 21‐1, and CA 125 were 0.0–5.0 ng/mL, 0.0–3.3 ng/mL, and 0.0–35.0 U/mL, respectively.

### Treatments

All patients underwent lobectomy or pneumonectomy. Mediastinal lymphadenectomy or systematic mediastinal lymph node sampling were also performed. Postoperative chemoradiotherapy was administered according to published recommendations following National Comprehensive Cancer Network guidelines.

### Endpoints

Overall survival (OS), progression‐free survival (PFS), local regional relapse‐free‐survival (LRFS), and distant metastasis‐free survival (DMFS) were observed as outcomes. Survival values were calculated from the time of resection. OS was calculated as the time to death. PFS was calculated as the time to documented clinical progression or death. LRFS was calculated as the time to local progression, defined as primary tumor or regional lymph node recurrence. DMFS was calculated as the time to distant metastasis.

### Serum tumor marker prognostic model

We built a prognosis model using the three serum tumor markers. Each abnormal marker was given one point. According to the scores, patients were divided into three groups: low‐risk (0 points), medium‐risk (1 point), and high‐risk (2–3 points).

### Statistical analysis

Survival values were calculated using the Kaplan‐Meier method. The cutoff value of each serum marker was the upper limit of its normal value (CEA: 5.0 mg/mL, CYFRA 21‐1: 3.3 ng/mL, and CA 125: 35.0 U/mL). Univariate analysis was performed using the log‐rank test to evaluate the association between each single marker and the survival outcomes. Multivariate analysis was performed using the Cox regression model; variables included sex, age, pathology, Karnofsky Performance Status (KPS), T stage, CEA, CYFRA 21‐1, CA 125, and postoperative treatments. Differences of characteristics and treatments among the three risk groups were evaluated using the chi‐squared test. Continuous variables are described as means; they were compared using Student's *t*‐test. If the chi‐squared test or *t*‐test showed differences in patients' clinical parameters or treatments, propensity score matching (PSM) was used (ratio 1:1:1) to decrease the bias. A *P*‐value of <0.05 was considered statistically significant. All statistical analyses were conducted using the SPSS statistical software package version 24.0 (SPSS Inc., Chicago, IL, USA).

## Results

### Patient characteristics

From March 2003 to September 2015, 1011 patients fulfilled the enrollment criteria, of which 653 were male. The median age was 57 (range: 25–80) years. Overall, 666 patients were diagnosed with adenocarcinoma, and 275 patients were diagnosed with squamous cell carcinoma. The median follow‐up time was 29.21 (range: 0.56–154.18) months. The details are presented in Table 1.

**Table 1:** Characteristics of all patients

		CEA			CYFRA 21‐1			CA 125		
N (%)	All	N	AN	*P*‐value	N	AN	*P*‐ value	N	AN	*P*‐ value
Number	1011	577	434		571	440		822	189	
Age (years)	57 (25–80)	57 (25–78)	58 (31–80)	0.559	57 (25–78)	58 (27–80)	0.278	58 (25–80)	55 (27–76)	0.385
<70	908 (89.8)	521 (90.2)	387 (89.2)		518 (90.7)	390 (88.6)		735 (89.4)	173 (91.5)	
≥70	103 (10.2)	56 (0.8)	47 (10.8)		53 (9.3)	50 (11.4)		87 (10.6)	16 (8.5)	
Sex				0.003			0.000			0.062
M	653 (64.6)	395 (68.5)	258 (59.4)		314 (55.0)	339 (77.0)		542 (65.9)	111 (58.7)	
F	358 (35.4)	182 (31.5)	176 (40.6)		257 (45.0)	101 (23.0)		280 (34.1)	78 (41.3)	
KPS				0.304			0.730			0.652
≥80	1003 (99.2)	571 (99.0)	432 (99.5)		566 (99.1)	437 (99.3)		815 (99.1)	188 (99.5)	
<80	8 (0.8)	6 (1.0)	2 (0.5)		5 (0.9)	3 (0.7)		7 (0.9)	1 (0.5)	
T stage				0.746			0.000			0.000
T1‐2	820	466 (80.8)	354 (81.6)		493 (86.3)	327 (74.3)		688 (83.7)	132 (69.8)	
T3‐4	191	111 (19.2)	80 (18.4)		78 (13.7)	113 (25.7)		134 (16.3)	57 (30.2)	
Pathology				0.000			0.000			0.015
Sq	275 (27.2)	213 (36.9)	62 (14.3)		89 (15.6)	186 (42.3)		237 (28.8)	38 (20.1)	
Nsq	736 (72.8)	364 (63.1)	372 (85.7)		482 (84.4)	254 (57.7)		585 (71.2)	151 (79.9)	
POChT				0.583			0.002			0.148
Yes	666 (65.9)	376 (65.2)	290 (66.8)		399 (69.9)	267 (60.7)		550 (66.9)	116 (61.4)	
No	345 (34.1)	201 (34.8)	144 (33.2)		172 (30.1)	173 (39.3)		272 (33.1)	73 (38.6)	
PORT				0.058			0.003			0.017
Yes	244 (24.1)	152 (26.3)	92 (21.2)		158 (27.2)	86 (19.5)		211 (25.7)	33 (17.5)	
No	767 (75.9)	425 (73.7)	342 (78.8)		413 (72.3)	354 (80.5)		611 (74.3)	165 (82.5)	

AN, abnormal; F, female; KPS, Karnofsky performance status M, male; N, normal; Nsq, nonsquamous carcinoma; POChT, postoperative chemotherapy; PORT, postoperative radiotherapy; Sq, squamous carcinoma.

### Patient characteristics of different risk groups

The low‐risk group consisted of 301 patients who received 0 points; 428 medium‐risk patients received one point; and of the 282 high‐risk patients, 211 received two points and 71 received three points. The low‐risk group had younger patients, more female patients, earlier T stages, more nonsquamous carcinomas, and a higher proportion of postoperative chemotherapy and radiotherapy compared with those in the medium‐ and high‐risk groups. After PSM, 226 patients were assigned to each group; age, sex, KPS scores, T stages, postoperative chemotherapy proportions, and postoperative radiotherapy portions were balanced among the three groups. The details are presented in Table 2.

**Table 2:** Patient characteristics of different risk groups

	Prognostic model of all points				Prognostic model after PSM			
N (%)	LR	MR	HR	*P*‐ value	LR	MR	HR	*P*‐ value
Number	301	428	282		226	226	226	
Age (years)	55 (25–78)	58 (30–78)	57 (27–80)	0.050	57 (25–78)	57 (30–78)	57 (27–76)	0.734
<70	278 (92.4)	373 (87.1)	257 (91.1)		204 (90.3)	203 (89.8)	207 (91.6)	
≥70	23 (7.6)	55 (12.9)	25 (8.9)		22 (9.7)	23 (10.2)	19 (8.4)	
Sex				0.039				0.254
M	177 (58.8)	284 (66.4)	192 (68.1)		142 (62.8)	157 (69.5)	143 (63.3)	
F	124 (41.2)	144 (33.6)	90 (31.9)		84 (37.2)	69 (30.5)	83 (36.7)	
KPS				0.619				0.477
≥80	298 (99.0)	424 (99.1)	281 (99.6)		223 (98.7)	225 (99.6)	225 (99.6)	
<80	3 (1.0)	4 (0.9)	1 (0.4)		3 (1.3)	1 (0.4)	1 (0.4)	
T stage				0.000				0.272
T1‐2	262 (87.0)	349 (81.5)	209 (74.1)		187 (82.7)	180 (79.6)	193 (85.4)	
T3‐4	39 (13.0)	79 (18.5)	73 (25.9)		39 (17.3)	46 (20.4)	33 (14.6)	
Pathology				0.000				0.187
Sq	63 (20.9)	143 (33.4)	70 (24.8)		61 (27.0)	79 (35.0)	70 (31.0)	
Nsq	239 (79.1)	285 (66.6)	212 (75.2)		165 (73.0)	147 (65.0)	156 (69.0)	
POChT				0.091				0.674
Yes	209 (69.4)	285 (66.6)	172 (61.0)		134 (59.3)	134 (59.3)	142 (62.8)	
No	92 (30.6)	143 (33.4)	110 (39.0)		92 (40.7)	92 (40.7)	84 (37.2)	
PORT				0.000				0.731
Yes	96 (31.9)	97 (22.7)	51 (18.1)		51 (22.6)	51 (22.6)	45 (19.9)	
No	205 (68.1)	331 (77.3)	231 (81.9)		175 (77.4)	175 (77.4)	181 (80.1)	

F, female; HR, high‐risk; KPS, Karnofsky performance status; LR, low‐risk; M, male; MR, medium‐risk; Nsq, nonsquamous carcinoma; POChT, postoperative chemotherapy; PORT, postoperative radiotherapy; Sq, squamous carcinoma.

### Prognostic significance of serum tumor markers

In univariate analysis, patients with normal CEA levels had higher values for five‐year OS (54.2% vs. 43.6%, *P* = 0.025), PFS (29.8% vs. 19.3%, *P* = 0.000), and DMFS (35.8% vs. 22.3%, *P* = 0.000) than patients with abnormal CEA levels. The five‐year LRFS was higher in the normal CEA group but without significant difference (40.2% vs. 33.1%, *P* = 0.057) (Fig S1a–d).

Patients with normal CYFRA 21‐1 levels had more favorable five‐year OS (55.6% vs. 43.4%, *P* = 0.001) and LRFS (40.6% vs. 32.8%, *P* = 0.000) than patients with abnormal levels. Five‐year PFS (25.9% vs. 25.7%, *P* = 0.218) and DMFS (30.3% vs. 30.6%, *P* = 0.310) were similar in patients with normal and abnormal CYFRA 21‐1 levels (Fig S2a–d).

Patients with normal CA 125 achieved higher five‐year OS (53.1% vs. 38.0%, *P* = 0.004), PFS (27.8% vs. 14.8%, *P* = 0.000), LRFS (40.3% vs. 24.5%, *P* = 0.000), and DMFS (32.3% vs. 21.0% *P* = 0.000) (Fig S3a–d) than patients with abnormal levels.

In multivariate analysis, CEA was associated with five‐year DMFS (*P* = 0.003) but not with five‐year OS (*P* = 0.171), PFS (*P* = 0.052), or LRFS (*P* = 0.408). CYFRA 21‐1 was associated with five‐year OS (*P* = 0.025) and LRFS (*P* = 0.023) but not with five‐year PFS (*P* = 0.165) or DMFS (*P* = 0.214). CA 125 was associated with all survival classifications (OS: *P* = 0.004, PFS: *P* = 0.005, LRFS: *P* = 0.004, DMFS: *P* = 0.027).

### Prognostic model

**Figure 1:**
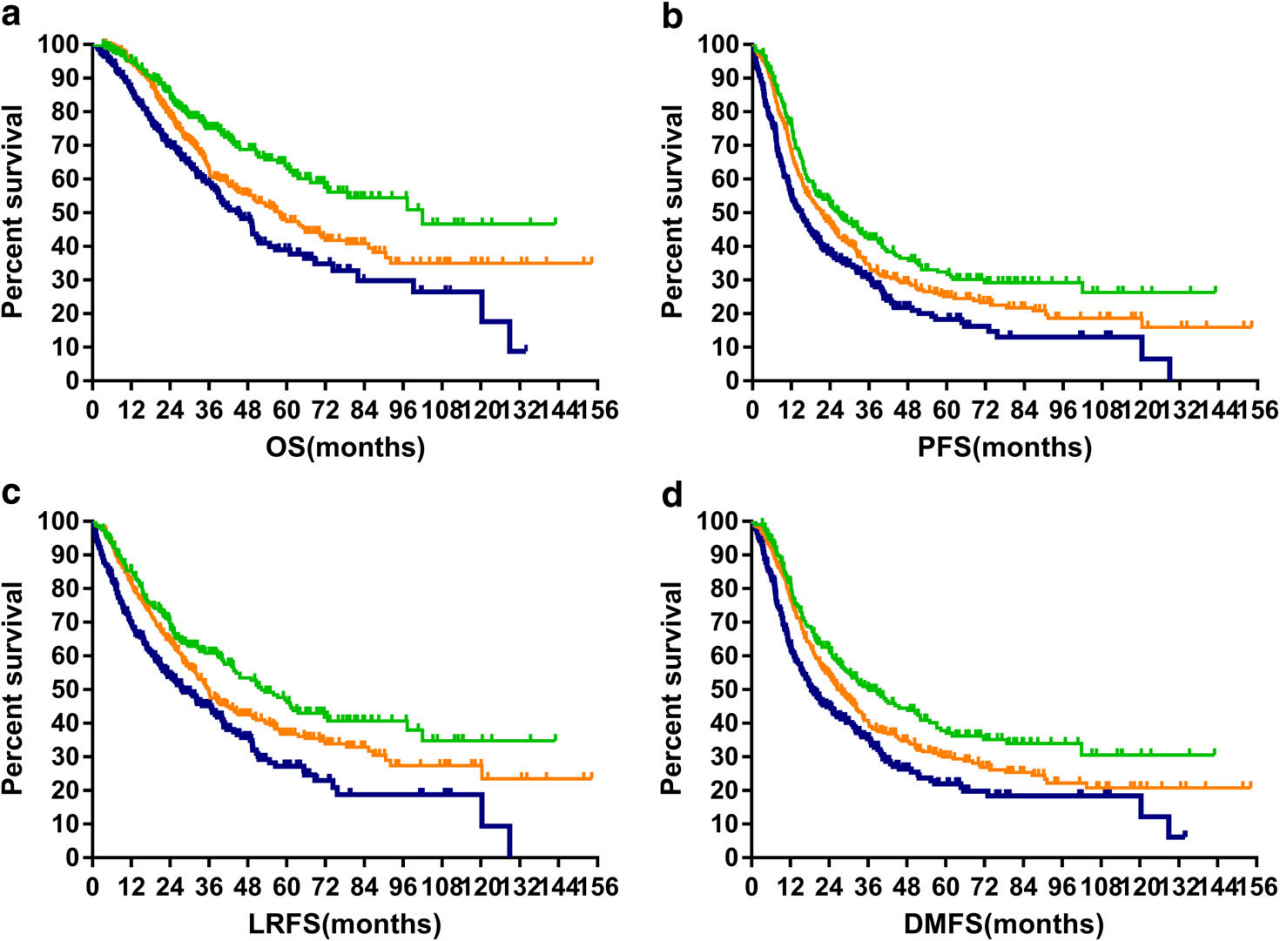
Survival of the different risk groups. (**a**) Overall survival (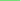) LR, (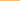) MR, (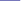) HR; (**b**) progression‐free survival (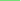) LR, (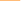) MR, (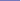) HR; (**c**) local regional relapse‐free survival (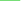) LR, (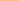) MR, (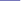) HR; and (**d**) distant metastasis‐free survival (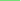) LR, (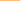) MR, (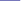) HR.

Among the 1011 patients, the five‐year OS for patients in the low‐, medium‐, and high‐risk groups were 63.9%, 48.0%, and 38.9%, respectively (*P* = 0.000). The corresponding results for PFS were 32.3%, 25.0%, and 18.0%, respectively (*P* = 0.000). This model also showed significant association with five‐year LRFS (47.7% vs. 36.7% vs. 27.2%, *P* = 0.000) and five‐year DMFS (37.8% vs. 30.0% vs. 22.0%, *P* = 0.000). The survival curves are plotted in Fig 1a–d.

**Figure 2:**
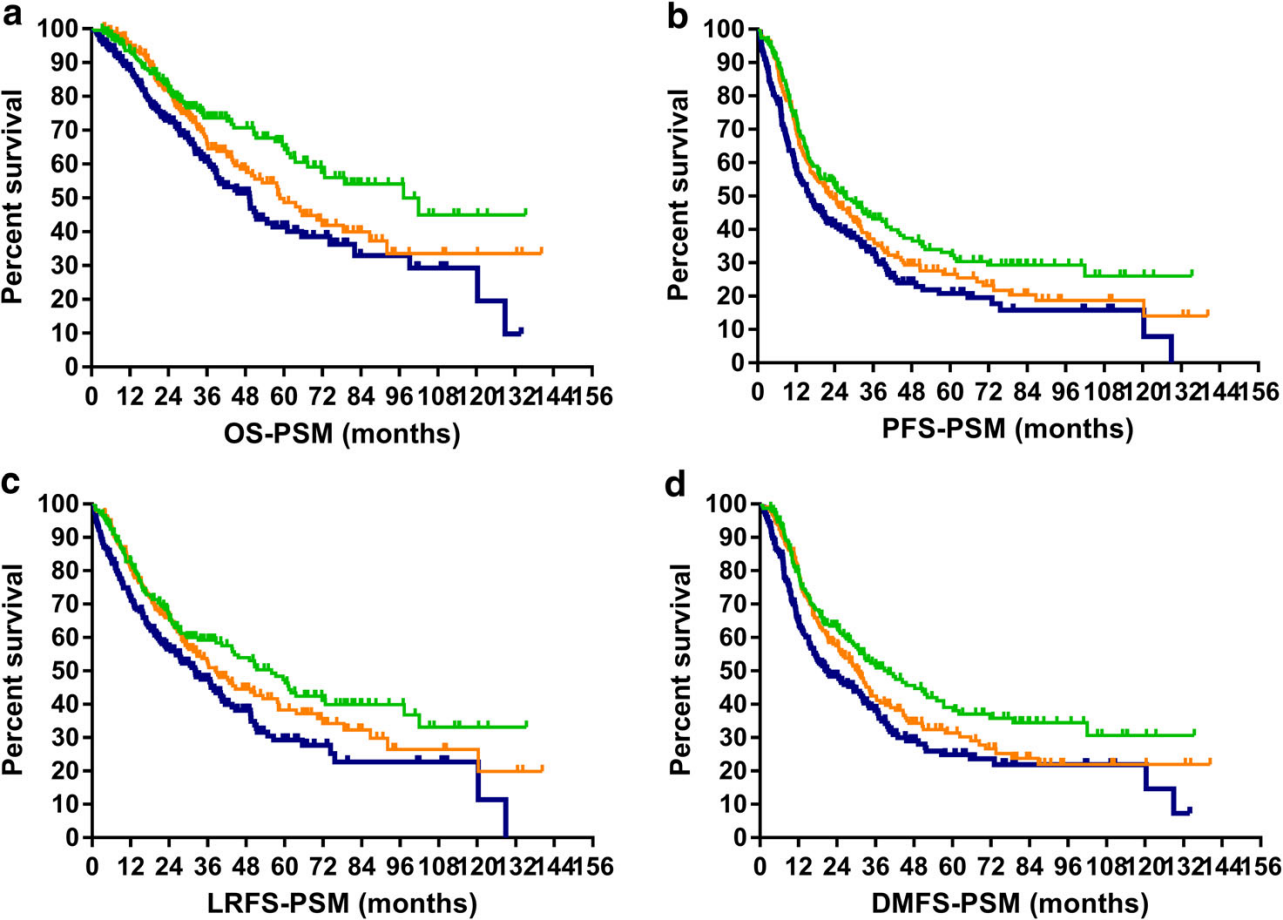
Survival of different risk groups after propensity score matching. (**a**) Overall survival (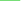) LR, (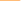) MR, (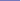) HR: (**b**) progression‐free survival (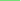) LR, (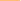) MR, (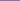) HR; (**c**) local regional relapse‐free survival (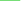) LR, (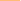) MR, (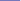) HR; and (**d**) distant metastasis‐free survival (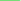) LR, (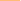) MR, (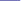) HR.

For the three groups of patients enrolled in the PSM analysis (low‐, medium‐, and high‐risk), the model achieved prognostic significance in all four survival classifications. Respectively, five‐year OS: 65.5% vs. 49.8% vs. 41.4%, *P* = 0.001; five‐year PFS: 33.1% vs. 26.5% vs. 20.8%, *P* = 0.002; five‐year LRFS: 47.4% vs. 38.3% vs. 29.3%, *P* = 0.004; five‐year DMFS: 39.1% v*s*. 31.4% vs. 24.9%, *P* = 0.001. The survival curves are plotted in Fig 2a–d.

## Discussion

Serum tumor markers are routinely examined for diagnosis and follow‐up. Although there are newer markers, classic serum tumor markers are very reliable, easily determined, economical, and highly reproducible. Relationships between elevated tumor marker serum levels and prognoses have been proposed, but these reports enrolled patients at different stages and with heterogeneous therapeutic regimens. Few studies have supplied information about locally advanced stages after surgery.^5^, ^6^, ^7^ Our study was only concerned with patients with N2 NSCLC after R0 resection.

CEA has been the most extensively studied tumor marker in lung cancer. However, definite conclusions have not been reported. Perioperative measurement of serum CEA concentration can yield information valuable for predicting poor survival; the pathological stages of these patients has been reported to be very heterogeneous (from IA to IV), which could induce bias.^8^ CEA has been shown to be a useful prognostic marker for OS and PFS by 18 trials, while it was not reliable in another seven trials.^9^ Biases including the cancer stage, period, different treatments, and a small number of patients might have contributed to this discrepancy. CEA was a good predictor of OS, PFS, and DMFS in univariate analysis in our study. However, in multivariate analysis, it was only associated with DMFS. The outcome of DMFS is in agreement with that in previous studies, which demonstrated that high levels of serum CEA might indicate a high risk of distant metastases.^10^

CYFRA 21‐1 is a useful tumor marker for NSCLC, and several studies have concluded that it is a significant prognostic determinant.^6^, ^11^ High levels of CYFRA 21‐1 have also been correlated with poor outcomes in squamous cell carcinoma.^12^ However, it has been reported to be a poor independent prognostic factor in surgically treated lung adenocarcinoma.^13^ Lower baseline CYFRA 21‐1 levels have also been associated with longer OS and PFS.^14^ Our study showed an association between CYFRA 21‐1 levels and OS, instead of PFS, both in univariate and multivariate analysis, and a relationship between LRFS and CYFRA 21‐1.

Compared with CEA and CYFRA 21‐1, there have been fewer studies on the relationship between CA 125 and survival. In operable NSCLC, serum CA 125 could be used as a tool to predict survival.^15^, ^16^, ^17^ A correlation between high baseline levels of CA 125 and worse survival in advanced stage (III–IV) NSCLC patients has been previously reported.^18^ However, it was not conclusive in predicting relapse and failures.^15^, ^17^, ^19^ Our study demonstrated that CA 125 is the only independent indicator for OS, PFS, LRFS, and DMFS, both in univariate and multivariate analyses.

CYFRA 21‐1 has been shown to be more sensitive than CEA in predicting OS;^20^ however, another study showed the predictive values are almost equal.^21^ In our study, CEA, CYFRA 21‐1, and CA 125 each had different advantages. CEA was a favorable prognostic factor of DMFS; CYFRA 21‐1 was a good predictor for LRFS; and CA 125 was a useful indicator of OS, PFS, LRFS, and DMFS.

Previous studies have used multiple tumor markers for modeling NSCLC prognosis. Patients with normal markers may have better PFS and OS than those with one or two high markers; but there were no differences reported in the study by Sone *et al*. in PFS and OS between patients with high‐CEA/normal‐CYFRA 21‐1 and those with normal‐CEA/high‐CYFRA 21‐1.^21^ Another model using CEA and CYFRA 21‐1 was only partially successful.^22^ Three other models, which combined CEA and CA 125, were built; however, the algorithms were complex and could not be conveniently calculated.^5^, ^23^, ^24^ Consequently, we devised a simple model to combine these three markers. Our model showed high predictive abilities for OS, PFS, LRFS, and DMFS.

Inevitably, imbalances existed among the risk groups, especially with regard to sex, T stage, pathology, and radiation. To neutralize the effects of these imbalances, we set up a PSM analysis. In PSM analysis, this model still worked well in predicting all four of the survival types. The results of our study agrees with another small trial that analyzed the prognostic value of CEA, CYFRA 21‐1, and CA 125 and showed that abnormal elevations of the three tumor markers worsened the prognosis.^18^

Expression levels of CEA, CYFRA 21‐1, and CA 125 have been shown to be linked with disease stage.^7^ For example, CEA levels have been shown to be associated with M stage.^10^ Patients who had increased preoperative levels of CYFRA 21‐1 presented with more extensive lymph node involvement (N2‐3).^25^, ^26^ Our study enrolled only N2M0 patients and compared T stage between normal and abnormal tumor marker groups. There were more patients with T3–4 stage disease in the abnormal CYFRA 21‐1 and CA 125 groups than in the normal tumor marker groups.

Some studies have indicated that CEA is a specific tumor marker of adenocarcinoma, which accounts for a large portion of nonsquamous cell cancer.^22^ Similarly, that CYFRA 21‐1 is specific for squamous carcinoma.^22^ Our study supports these results. Most patients in the CEA abnormal group had a higher ratio of nonsquamous carcinoma than those in the CEA normal group. In contrast, the abnormal CYFRA 21‐1 group had more squamous carcinoma patients than the normal CYFRA 21‐1 group.

Cutoff values are another concern. Previous studies usually defined their own cutoff values after receiver operating characteristic analysis. CEA cutoff values were 2.5–50 ng/mL^5^, ^8^, ^9^, ^11^, ^15^, ^27^, ^28^ CYFRA 21‐1, 1.95–18 ng/mL;^5^, ^6^, ^7^, ^11^, ^12^, ^25^, ^26^, ^27^, ^29^ and CA 125, 15–100 U/mL.^15^, ^16^, ^18^, ^19^ All these studies did not support the cutoff values of other studies. Therefore, it is challenging to reach a widely accepted and easily repeated standard. Therefore, in this study, we used the cutoff values used in diagnosis, which were convenient and efficient.

The advantages of this study were multifold. It had a large sample size, and is the first study to investigate the prognostic prediction of N2 NSCLC patients after resection using serum tumor markers. Moreover, the prognosis model also proved to be useful and sensitive after multivariate PSM analysis. Previous models were useful for just one endpoint; this combined model was predictive for four classes: survival, recurrence, progression, and metastasis. The model in our study also utilized available technology, conventional cutoff values, and simple mathematics methods and was economical.

Our study had certain limitations. Other NSCLC tumor markers such as NSE and SCC were not analyzed, which might affect survival in certain pathological types such as neuroendocrine NSCLC and squamous carcinoma. We did not investigate the baseline trends and the values after treatment, which might also be helpful in predicting outcomes and has been evaluated in other NSCLC stages.^30^ Meanwhile, more than 30% of patients did not receive adjuvant chemotherapy, and nearly 80% of patients did not receive adjuvant radiation therapy, despite the fact that postoperative chemoradiation is recommended for stage III‐N2 NSCLC in the current version of the NCCN guidelines. The absence of adjuvant chemoradiotherapy would affect the prognosis to some extent. The reasons for this are multifold. As we know, until now there has been a lack of strong evidence for the actual benefit, especially overall survival from postoperative radiotherapy. The results from different studies are inconsistent and the postoperative radiation group had an even worse prognosis in some studies. Therefore, adjuvant radiotherapy is not given routinely and some patients even reject treatment with radiotherapy due to the potential risk of toxicities.^31^ Similarly, there has also been no strong evidence for the utilities of postoperative chemotherapy for a long time until the ANITA study was published in 2006.^32^ Patients diagnosed before 2006 did not regularly receive postoperative chemoradiation. Moreover, validation of the model in further research is also warranted.

In conclusion, the serum levels of CEA, CYFRA 21‐1, and CA 125 are prognostic predictors of stage N2 patients who have received R0 resection. The three‐marker combination model accurately predicts multiple prognostic outcomes (OS, PFS, LRFS, and DMFS), which might be helpful when deciding on future adjuvant therapy.

## Disclosure

Yirui Zhai, Zhouguang Hui, Yu Men, Yang Luo, Yushun Gao, Jingjing Kang, Xin Sun, and Jianyang Wang each declare that they have no competing interests.

## Supporting information


**Figure S1** Survival of patients with different CEA levels: (a) overall survival, (b) progression‐free survival, (c) local regional relapse‐free survival, (d) distant metastasis‐free survival

**Figure S2** Survival of patients with different CYFRA 21‐1 levels: (a) overall survival, (b) progression‐free survival, (c) local regional relapse‐free survival, (d) distant metastasis‐free survival.

**Figure S3** Survival of patients with different CA 125 levels: (a) overall survival, (b) progression‐free survival, (c) local regional relapse‐free survival, (d) distant metastasis‐free survival.
