# Supplementary figures and images for: Combined neat model for the prognosis of postoperative stage III‐N2 non‐small cell lung cancer
Source: Thorac Cancer. 2020 Jul 29;11(9):2610–7. doi: 10.1111/1759-7714.13585 (PMC7471036; doi:10.1111/1759-7714.13585)

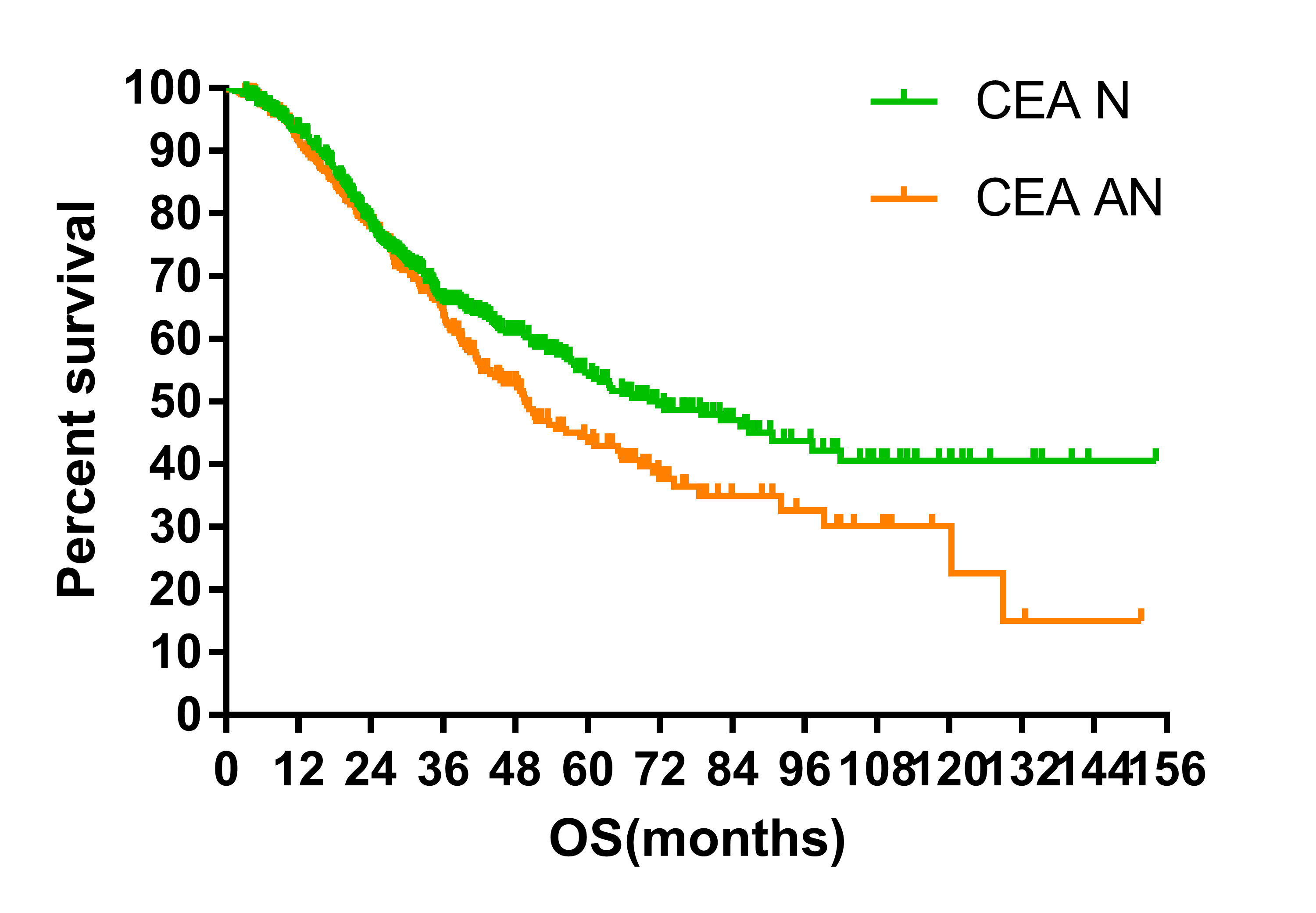

Supplement: Supplementary file 1 — Figure S1 Survival of patients with different CEA levels: (a) overall survival, (b) progression‐free survival, (c) local regional relapse‐free survival, (d) distant metastasis‐free survival [file TCA-11-2610-s001.zip › TCA_13585_s1a.tif]

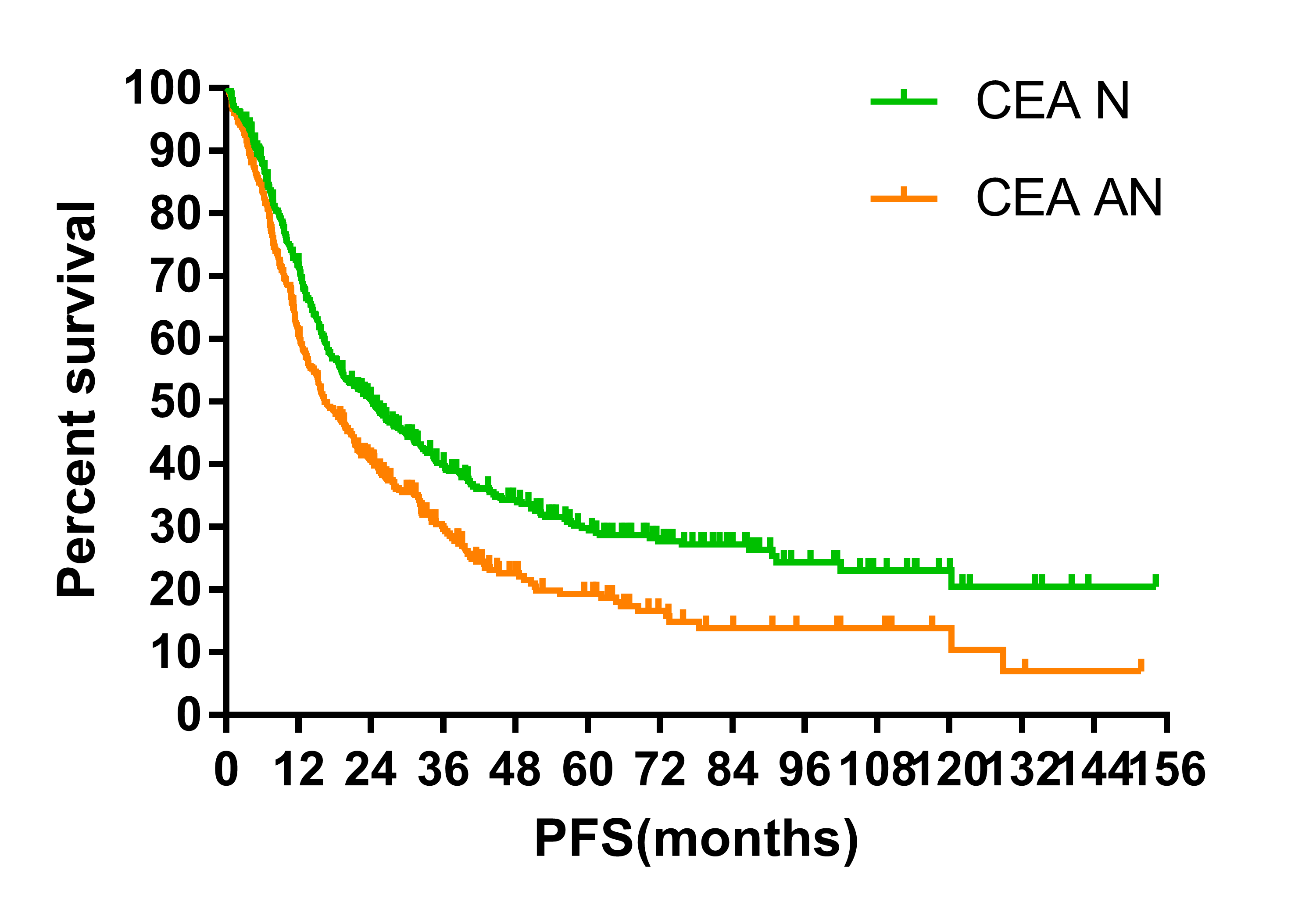

Supplement: Supplementary file 1 — Figure S1 Survival of patients with different CEA levels: (a) overall survival, (b) progression‐free survival, (c) local regional relapse‐free survival, (d) distant metastasis‐free survival [file TCA-11-2610-s001.zip › TCA_13585_s1b.tif]

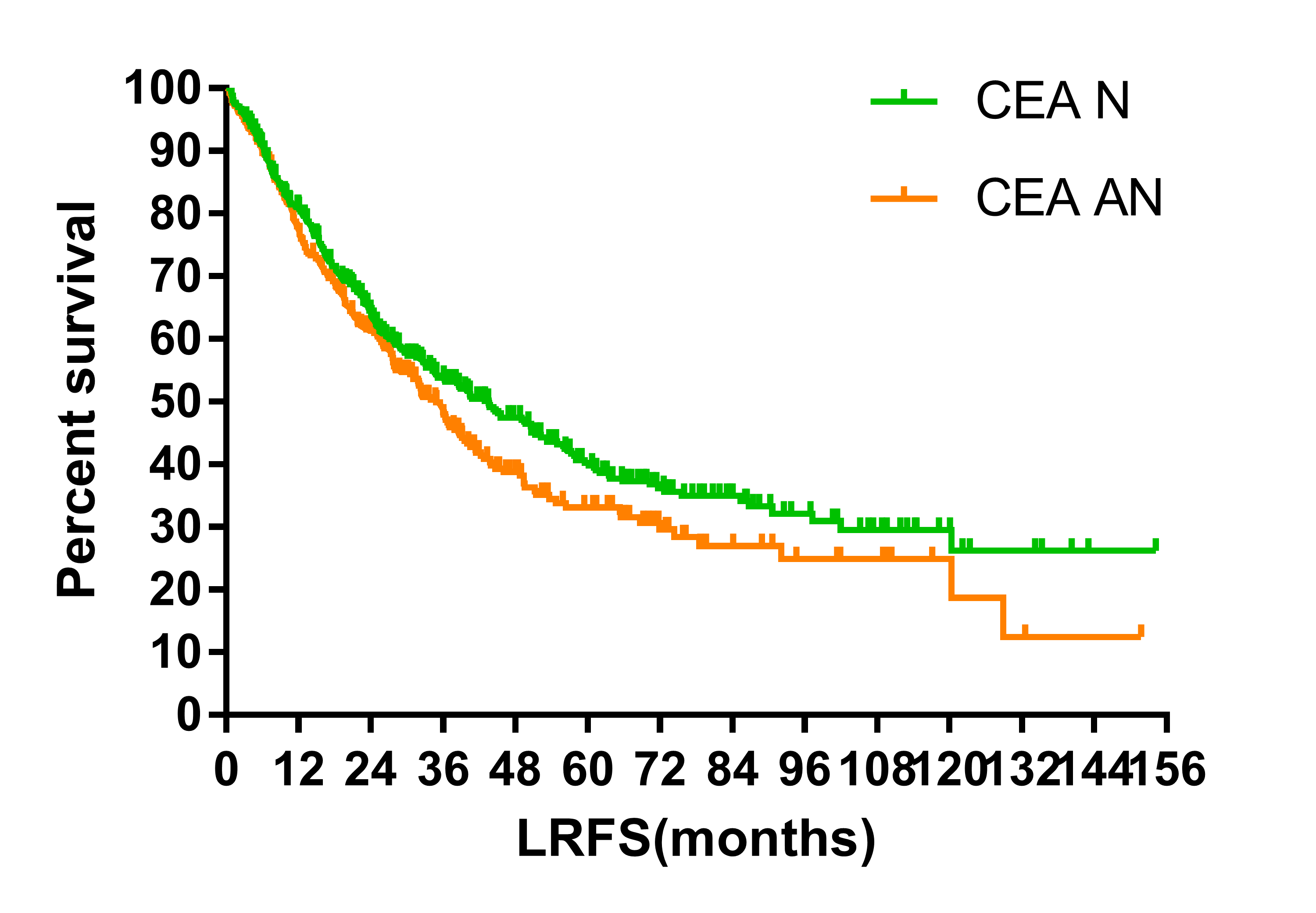

Supplement: Supplementary file 1 — Figure S1 Survival of patients with different CEA levels: (a) overall survival, (b) progression‐free survival, (c) local regional relapse‐free survival, (d) distant metastasis‐free survival [file TCA-11-2610-s001.zip › TCA_13585_s1c.tif]

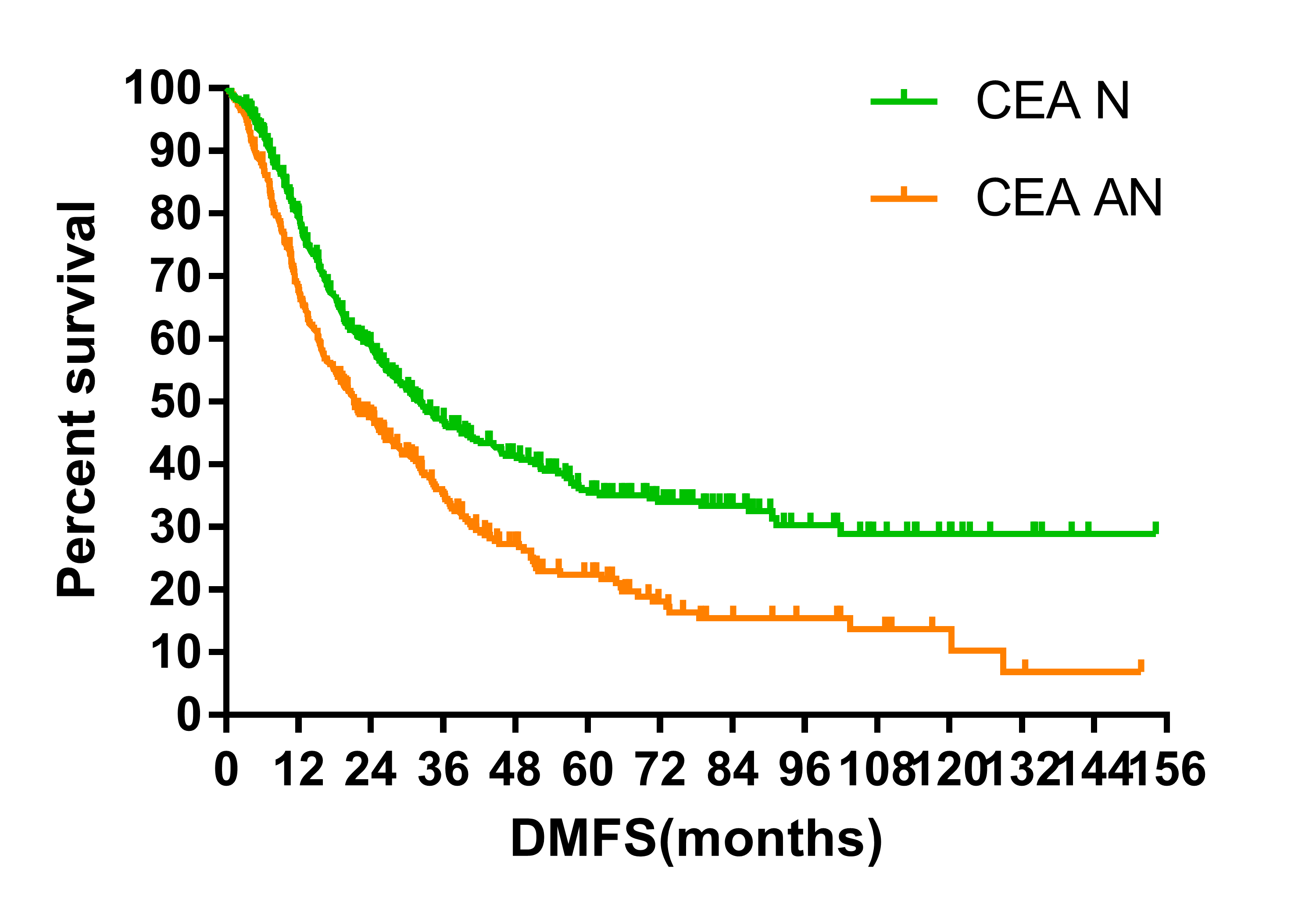

Supplement: Supplementary file 1 — Figure S1 Survival of patients with different CEA levels: (a) overall survival, (b) progression‐free survival, (c) local regional relapse‐free survival, (d) distant metastasis‐free survival [file TCA-11-2610-s001.zip › TCA_13585_s1d.tif]

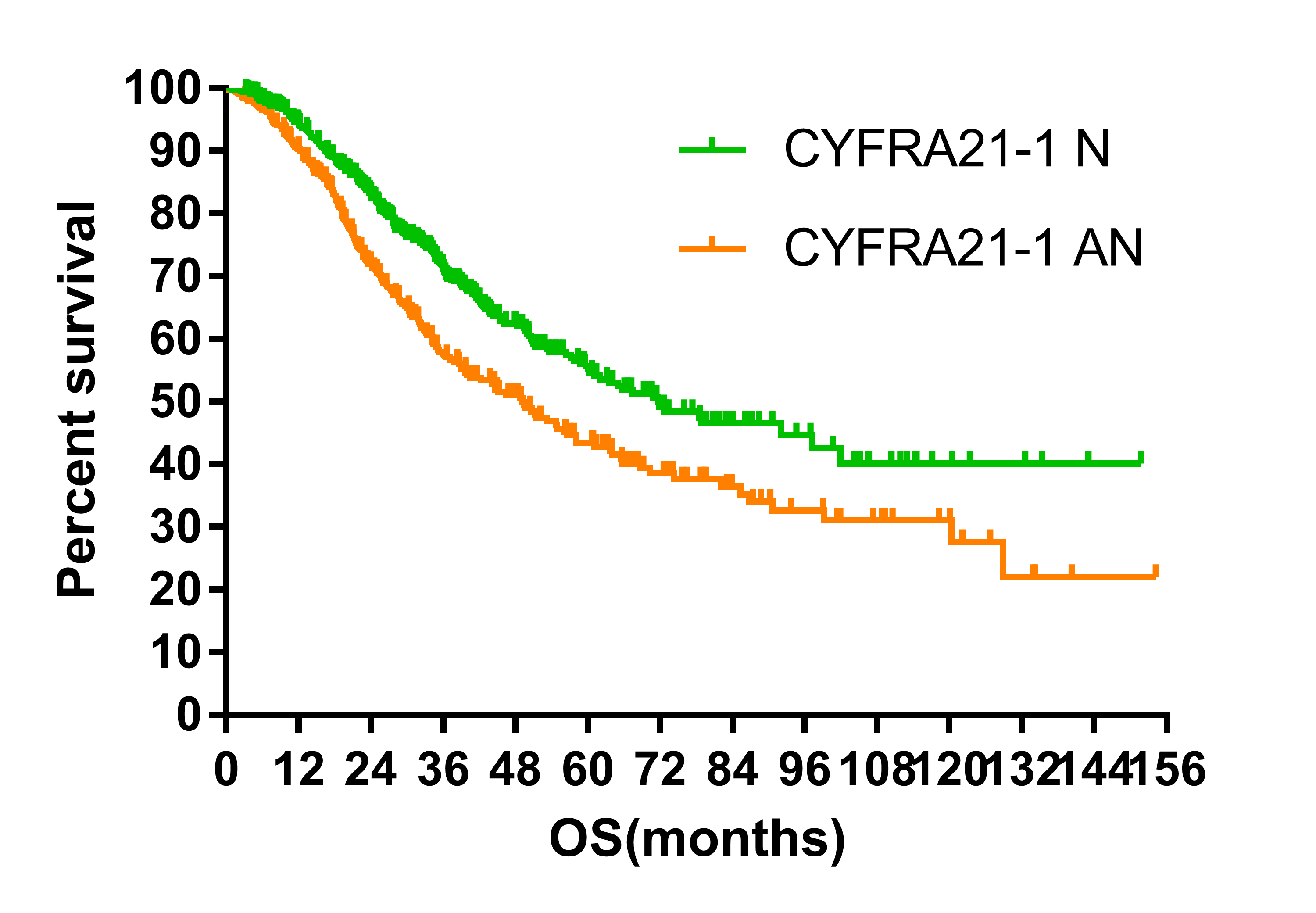

Supplement: Supplementary file 2 — Figure S2 Survival of patients with different CYFRA 21‐1 levels: (a) overall survival, (b) progression‐free survival, (c) local regional relapse‐free survival, (d) distant metastasis‐free survival. [file TCA-11-2610-s002.zip › TCA_13585_s2a.tif]

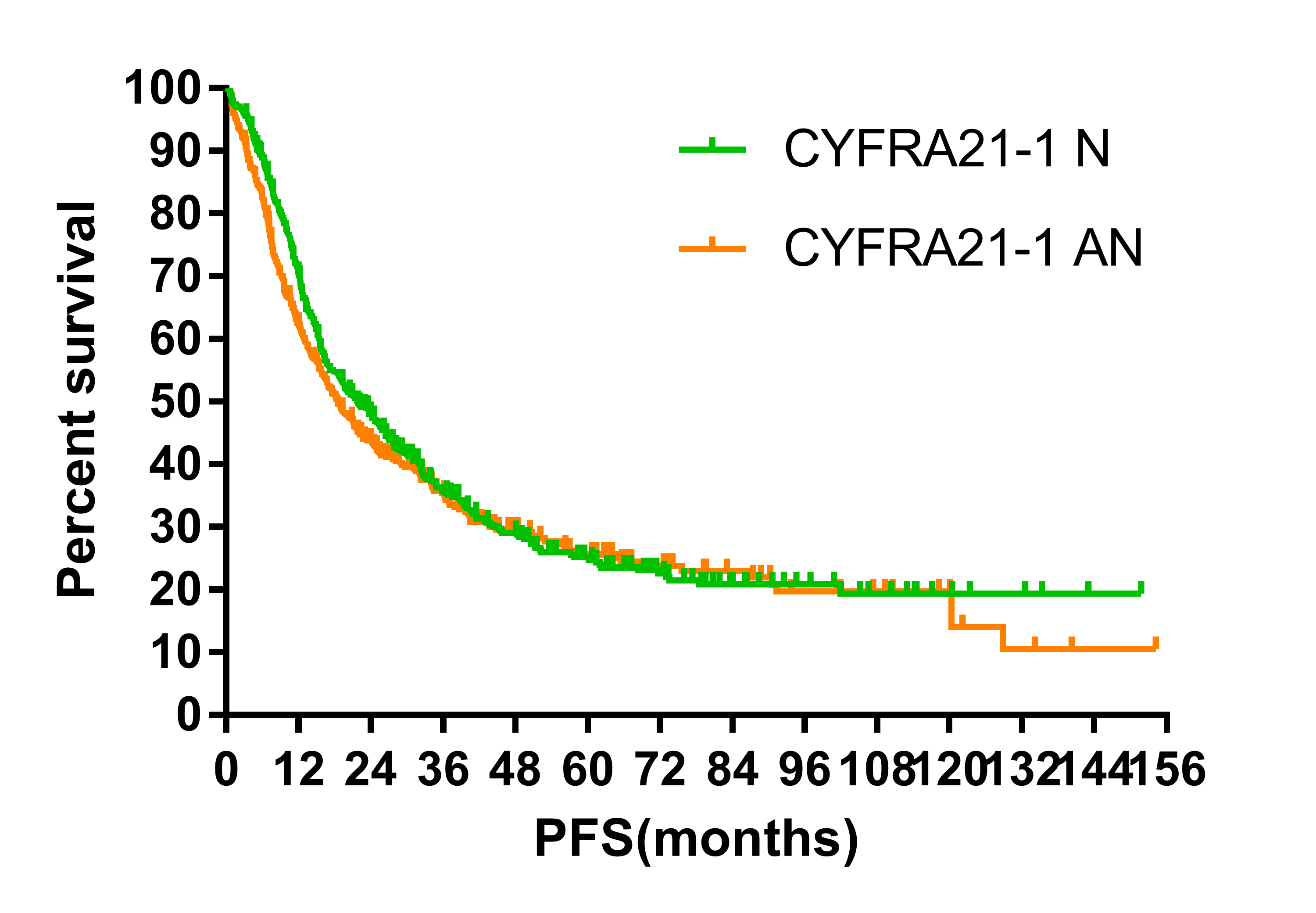

Supplement: Supplementary file 2 — Figure S2 Survival of patients with different CYFRA 21‐1 levels: (a) overall survival, (b) progression‐free survival, (c) local regional relapse‐free survival, (d) distant metastasis‐free survival. [file TCA-11-2610-s002.zip › TCA_13585_s2b.tif]

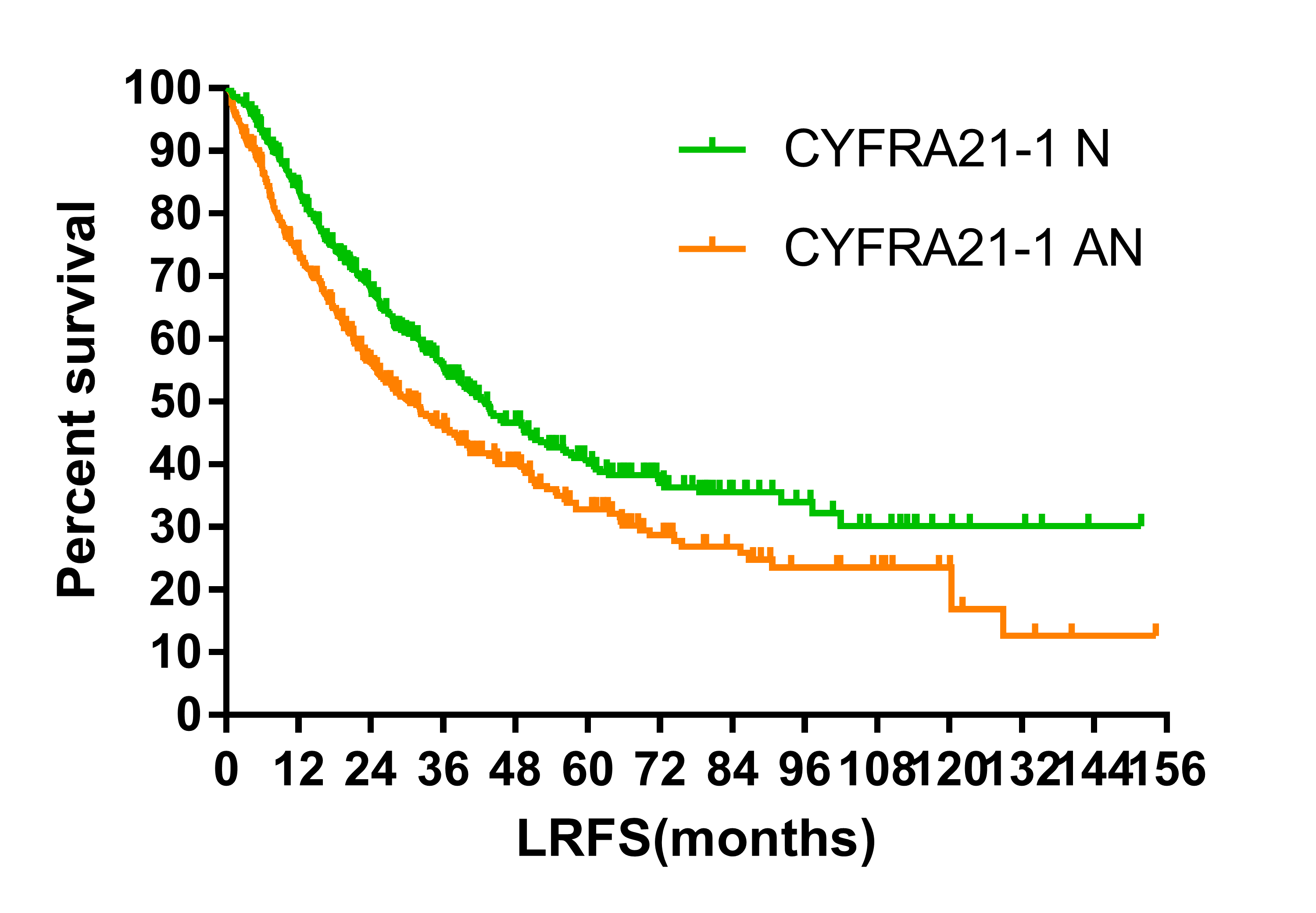

Supplement: Supplementary file 2 — Figure S2 Survival of patients with different CYFRA 21‐1 levels: (a) overall survival, (b) progression‐free survival, (c) local regional relapse‐free survival, (d) distant metastasis‐free survival. [file TCA-11-2610-s002.zip › TCA_13585_s2c.tif]

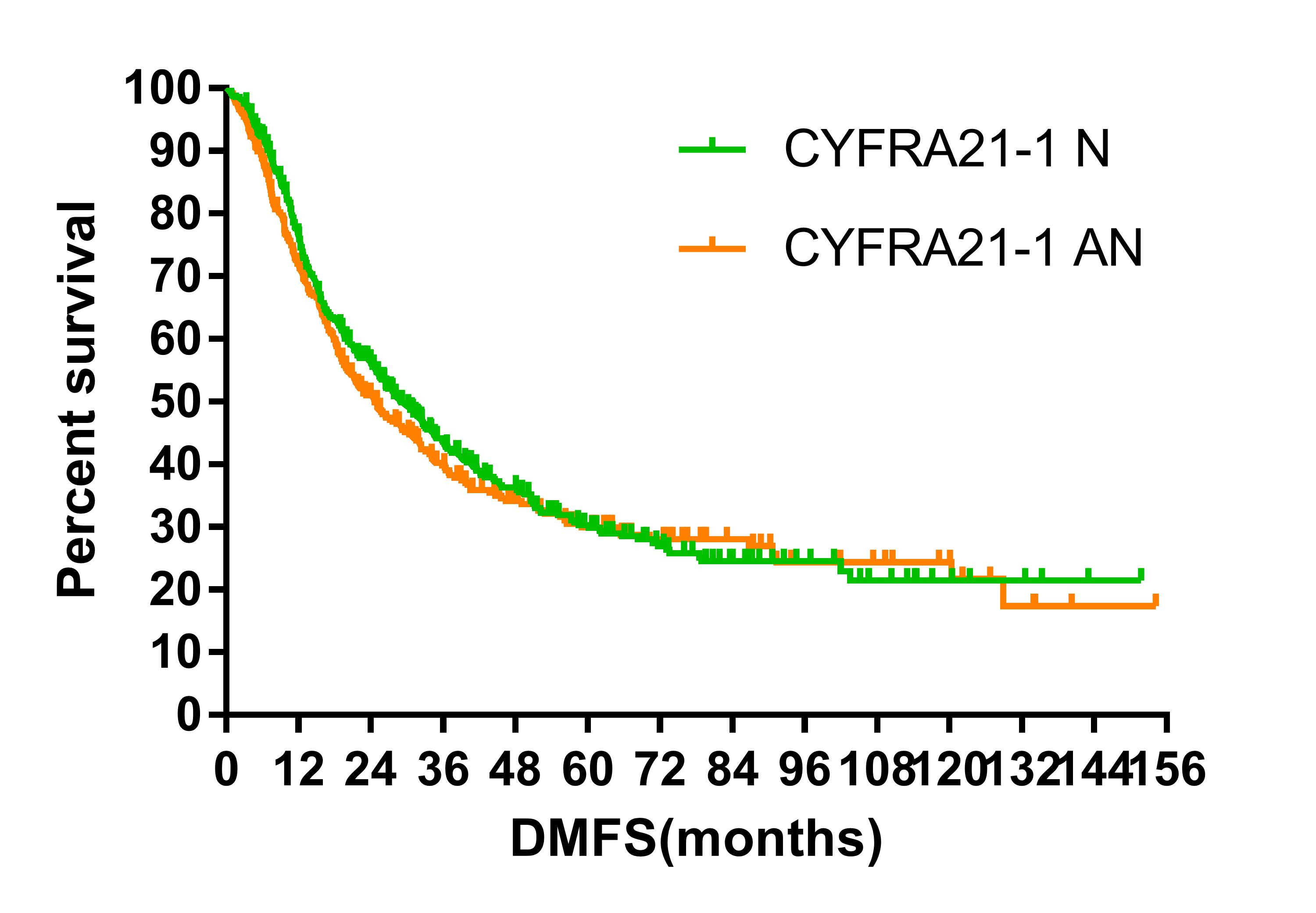

Supplement: Supplementary file 2 — Figure S2 Survival of patients with different CYFRA 21‐1 levels: (a) overall survival, (b) progression‐free survival, (c) local regional relapse‐free survival, (d) distant metastasis‐free survival. [file TCA-11-2610-s002.zip › TCA_13585_s2d.tif]

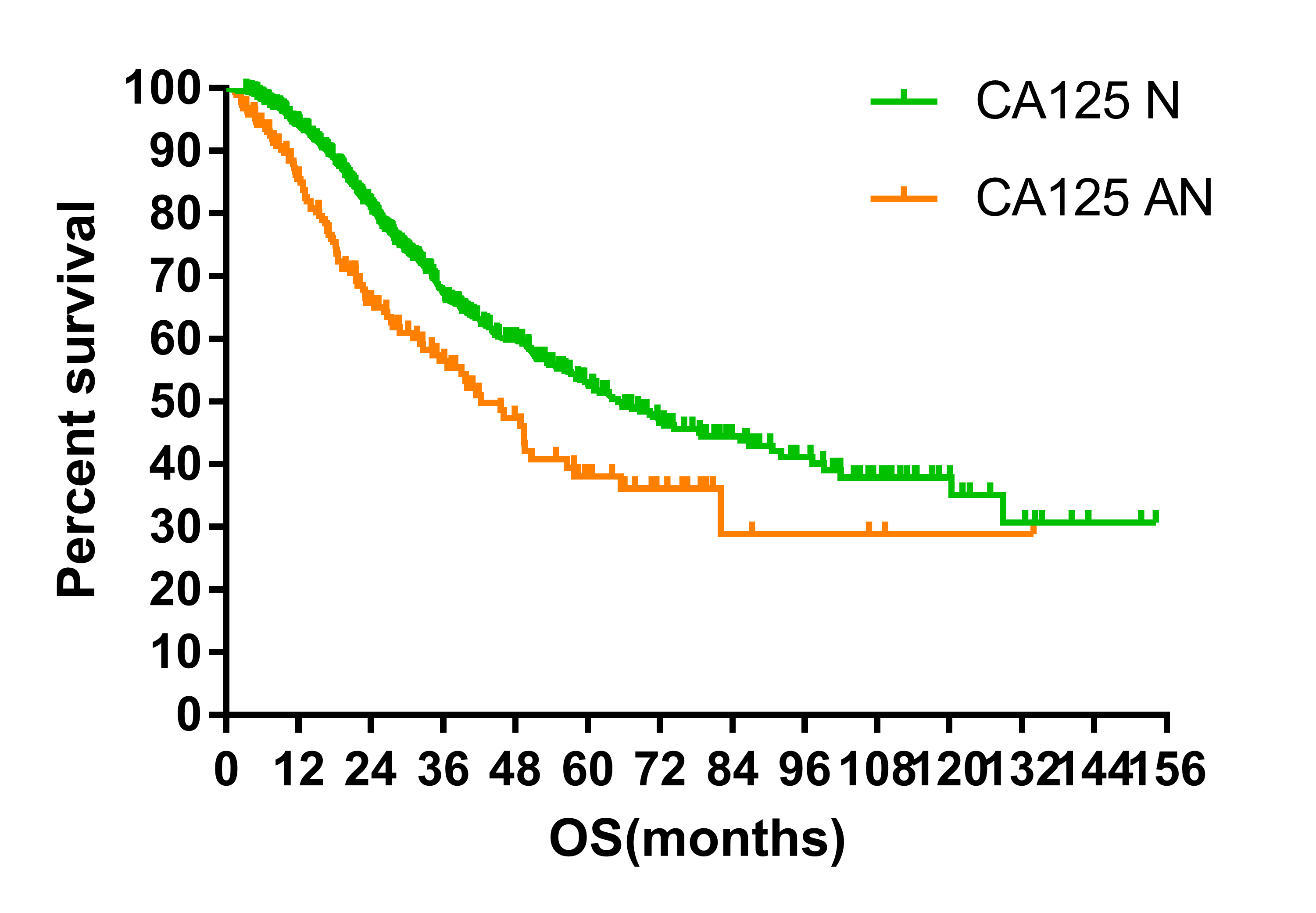

Supplement: Supplementary file 3 — Figure S3 Survival of patients with different CA 125 levels: (a) overall survival, (b) progression‐free survival, (c) local regional relapse‐free survival, (d) distant metastasis‐free survival. [file TCA-11-2610-s003.zip › TCA_13585_s3a.tif]

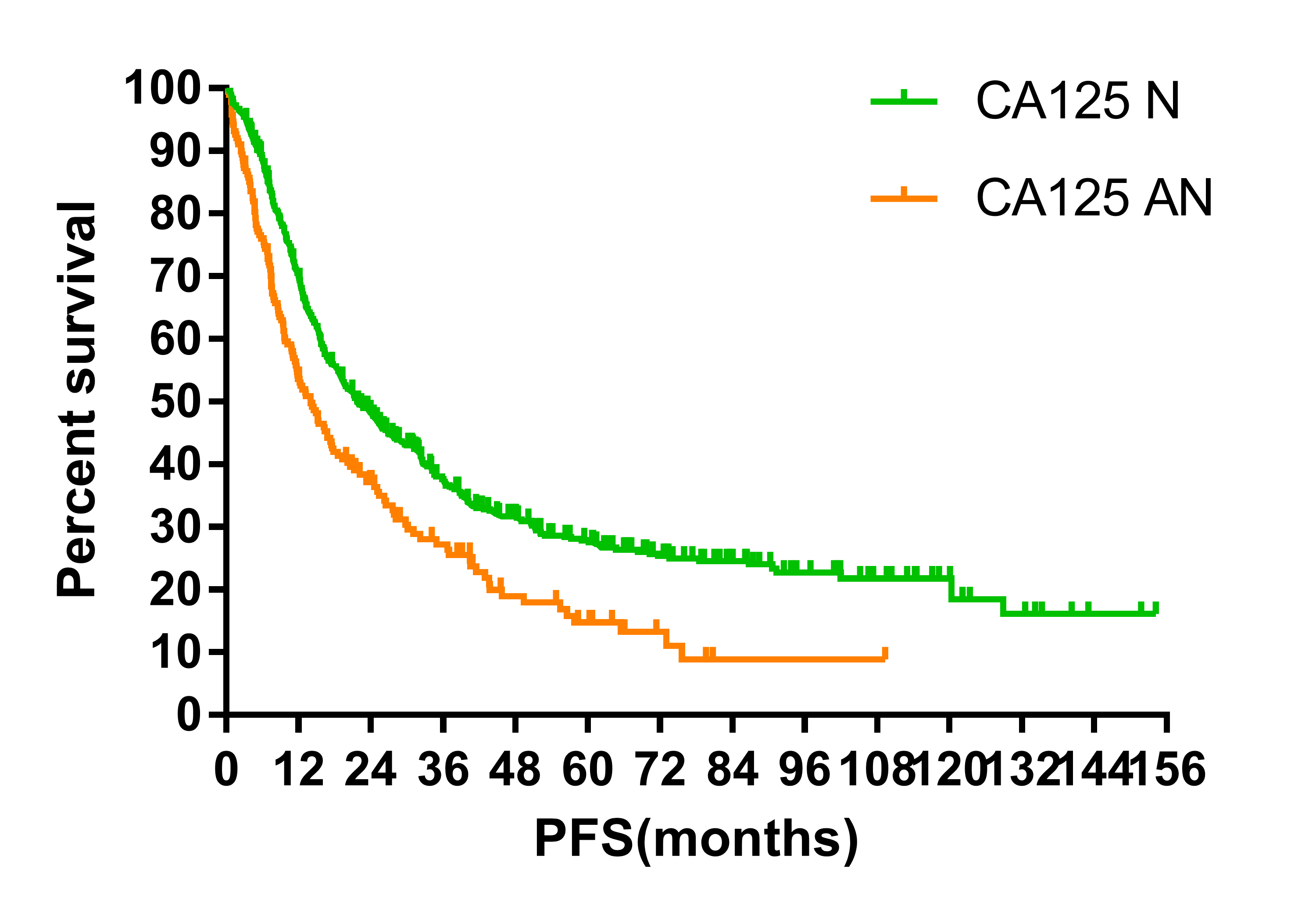

Supplement: Supplementary file 3 — Figure S3 Survival of patients with different CA 125 levels: (a) overall survival, (b) progression‐free survival, (c) local regional relapse‐free survival, (d) distant metastasis‐free survival. [file TCA-11-2610-s003.zip › TCA_13585_s3b.tif]

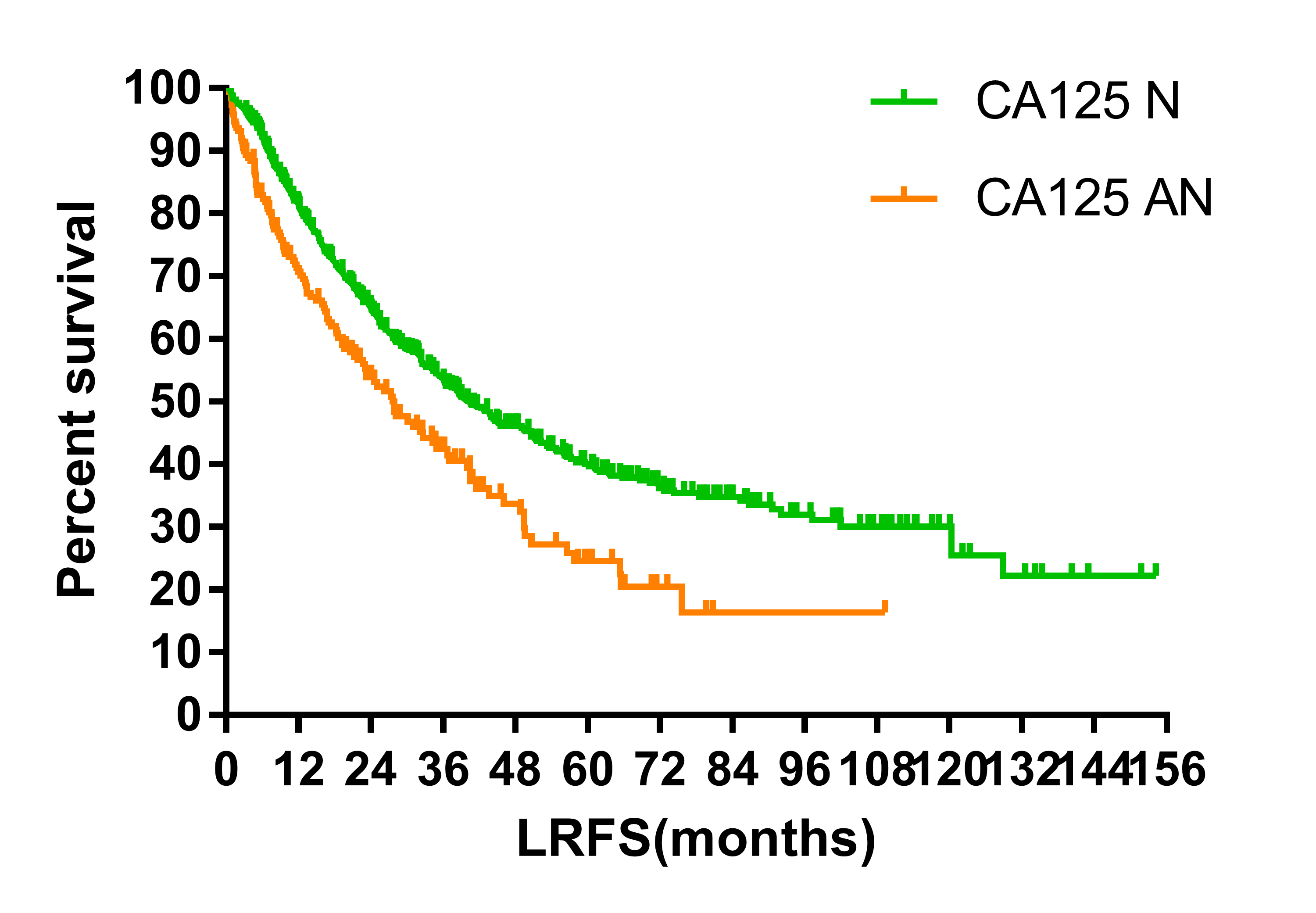

Supplement: Supplementary file 3 — Figure S3 Survival of patients with different CA 125 levels: (a) overall survival, (b) progression‐free survival, (c) local regional relapse‐free survival, (d) distant metastasis‐free survival. [file TCA-11-2610-s003.zip › TCA_13585_s3c.tif]

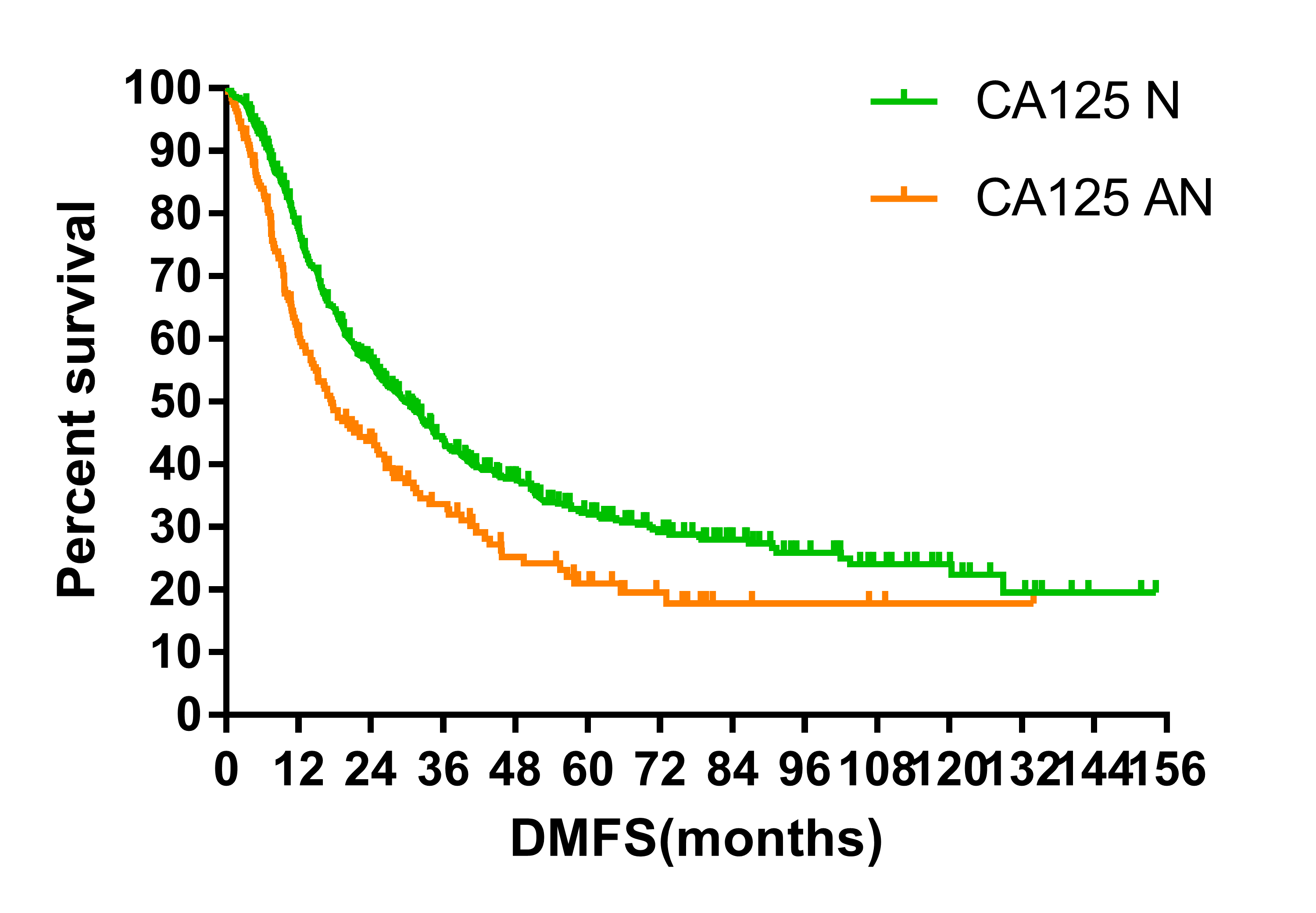

Supplement: Supplementary file 3 — Figure S3 Survival of patients with different CA 125 levels: (a) overall survival, (b) progression‐free survival, (c) local regional relapse‐free survival, (d) distant metastasis‐free survival. [file TCA-11-2610-s003.zip › TCA_13585_s3d.tif]
